# Interpersonal behaviors and socioemotional interaction of medical students in a virtual clinical encounter

**DOI:** 10.1186/1472-6920-14-64

**Published:** 2014-04-01

**Authors:** Olivier Courteille, Anna Josephson, Lars-Olof Larsson

**Affiliations:** 1 Department of Learning, Informatics, Management & Ethics (LIME), Karolinska Institutet, Stockholm, Sweden; 2 Department of Neuroscience, Karolinska Institutet, Stockholm, Sweden; 3 Division of Respiratory Medicine, Department of Medicine, Karolinska University Hospital, Stockholm, and Angered Hospital, Gothenburg, Sweden

**Keywords:** Virtual clinical encounter, Affective learning, Computer-mediated interpersonal communication, Emotional engagement, socio-emotional interaction

## Abstract

**Background:**

The virtual clinical encounter (VCE) may function as an important support for medical students in or prior to clinical practice to train and ease communication and socioemotional interactions with patients*.* Few studies have however focused on the dynamics of interpersonal behaviors in clinical interviewing with a virtual patient (VP) and the affective responses evoked by such a learning experience. The study was designed to investigate the dynamics and congruence of interpersonal behaviors and socioemotional interaction exhibited during the learning experience in a VCE, and to evaluate which interaction design characteristics contribute most to the behavioral and affective engagement in medical students.

**Methods:**

Thirty medical students (sixth semester) participated voluntarily in an exploratory observational study with a highly interactive VP case based on a trustworthy VP encounter with a natural and realistic dialogue interface. Students worked collaboratively in pairs. They were videotaped for further behavioral analysis and self-reported (in both a survey and an interview) their personal opinions, perceptions and attitudes about the VCE. A mixed methods approach was applied.

**Results:**

All participants demonstrated an adequate, respectful and relevant clinical case management and to obtain psychosocial history. The collaborative workspace played its role and led to dynamic and engaged discussions fostering thus shared understanding. The results suggest that the VCE studied was perceived as a meaningful, intrinsically motivational and activating learning environment, and was found to socially and emotionally engage learners. We also found that VCEs have the potential to support the development of relevant and congruent interpersonal communication skills in trainees.

**Conclusions:**

By taking advantage of socioemotional interaction, VCEs promote not only critical reflection skills or strategy-selection skills, but also to develop listening and nonverbal skills, induce self-awareness and target coping behaviours. We believe that, if applied in early medical education, this learning approach may facilitate clinical encounters at an early stage and contribute to responsible clinical decision making.

## Background

Medical students do not only need to acquire knowledge and skills to have a sound scientific basis for practicing medicine, but need to develop verbal and nonverbal communication skills as well (Bullimore [1]; Lloyd & Bor [2]). Furthermore, it is argued that proficient interpersonal interviewing skills in history taking are crucial to clinical learning (van Dulmen & Holl [3]) and for supporting and developing patient trust and reducing the person’s anxiety (O’Sullivan [4]). Subsequently, lack of proficiency in communication skills between the doctor and the patient may cause misunderstanding and delay of diagnosis since for example there is no firm clear-cut diagnosis at many of the first encounters. Although, the potency of interpersonal communication skills is considered as essential in critical clinical situations, in particular in the hospital staff teamwork; a Swedish study, for example, revealed an uncertainty in these kinds of situations among doctors in residency training (Hoppé [5]).

However, appropriate and effective training in specific communication skills is not always delivered at an early stage of medical education and many clinicians develop their interpersonal skills on the job (Kramer *et al*. [6]; Lundine *et al*. [7]; Wouda & van de Wiel [8]). The use of virtual learning environments favoring experiential learning and role modeling, such as Virtual Clinical Encounters (VCEs), was suggested to supply more targeted individualized training in clinical interviewing skills. VCEs, featured by Virtual Patients (VPs), are advanced, contextualized and simulated learning environments which portray real-life clinical encounters and have already been proven efficient and cost-effective complementary educational tools in healthcare (Cook & Triola [9]).

From an educational perspective, learning by encounter—either real or virtual—involves the affective mode of learning (primarily emotions and feelings) and presents demonstrated pedagogical benefits (Roberts *et al*. [10]; Rager [11]). A number of studies have shown the strong impact of socioemotional learning on cognitive learning outcomes, leading in particular to enhanced concentration, attention, motivation, understanding and knowledge retention (Sweller [12]; Sansone & Morgan [13]; Elias *et al.*[14]; Zins *et al.*[15]), including the functional relevance of emotions for engagement and achievement of the students (special issue by Linnenbrink-Garcia & Pekrun [16]).

Furthermore, it is important to be aware of the effects of multisensory integration on emotional engagement (Quinn [17]). According to Moreno [18], presenting information in several sensory modalities (by combining the human selective attention in hearing, vision and touch) leads to a more efficient use of memory resources. We can thus easily conceive the potential educational benefits of multimedia technology using multisensory presentation of graphic audio and visual materials. The same applies to the importance of naturalness of visual and auditory interaction (such as interactive video) for the sense of mediated presence. This latter is one of the key factors for emotionally mediated experiences (Picard [19], Johnson *et al*. [20]) and characterized by high levels of arousal and intensive affect (Dede *et al.*[21]). Knowing that emotional reactions and presence are highly correlated in experiential learning, we can envision the potential of their synergy in the accuracy of recalling information.

Prospective studies in cognitive psychology have thoroughly analyzed elicited emotions in relation to psychosocial and neuropsychological factors (Mayer [22]; Mayer & Gaschke [23]; Mayer *et al.*[24]; Revelle [25]). They emphasize that interpersonal judgment relies mostly on nonverbal and appearance cues from the social interaction partner. These aspects include various human traits, including thoughts, emotions, gestures, motivations and attitude (Salovey & Mayer [26]; Gross & John [27]).

Johnsen *et al.*[28] observed that interpersonal interaction skills used with a virtual human translated to the interaction skills used with a real human. In other words, the acquired interviewing skills are congruent in the real world. Moreover, experimental studies have actually indicated that Patient Simulation featured with a virtual conversation interface in natural language provides not only a higher degree of realism, but also a more profound learning experience (Stevens *et al.*[29]; Deladisma *et al.*[30]). Unfortunately, few studies have explored the affective dimension in training with VPs (Bearman & Cesnik [31]; Bearman [32]; Baños *et al*. [33]). Along with the imbalance between affect and cognition, such heuristics seem to have been neglected, and most of the focus in VP research has instead been on usability, clinical reasoning and assessment issues (Cook and Triola [9]). There is a need to attain a deeper insight on the dynamics of interpersonal behaviors and socioemotional interaction during the learning experience in a virtual patient encounter. Such information is also important to design more authentic, socioemotionally engaging, and meaningful learning interactions between students and VPs.

### Aims of the study

A primary aim was to investigate the dynamics and congruence of interpersonal behaviors and socioemotional interaction exhibited during the learning experience in a VCE. A secondary aim was to evaluate which interaction design characteristics contribute most the behavioral and affective engagement in medical students.

Our main research questions included the following:

○ Do trainees exhibit congruent interpersonal behaviors and relevant socioemotional engagement in a virtual clinical encounter?

○ Can the affective responses of the trainees evoked during the VCE learning experience possibly have a positive impact on learning and cognitive achievement?

More specific research questions included:

○ What kind of verbal and nonverbal cues can be observed in this kind of computer-mediated communication?

○ In a VCE, what are the key design characteristics that contribute most to behaviorally and affectively engage trainees?

## Methods

### Rationale for the used VP environment

The Interactive Simulation of Patients (ISP), developed at Karolinska Institutet, is a virtual patient simulation technology that builds on the engagement-education synergy, and leads to the convergence of information processing (clinical data), interactive communication and media technologies (Bergin & Fors [34]). ISP has been designed to simulate realistic patient encounters presented in a natural (life-like) interface. Students have at their disposal an interactive free-text driven patient-history function in natural language (supporting both Swedish and English), with all patient responses available in the form of pre-recorded video clips of actors simulating different patient cases. The medical history interface is designed not only to react to asked questions, but also to trigger affective reactions such as irritation, anger, fear, etc., with the VP depending on how questions are phrased, and whether they are repeated or inappropriate. The collaborative workspace of the ISP system also facilitates interaction among students, as well as between students and teachers/facilitators (Bergin *et al*. [35]). These intrinsic characteristics led us to choose ISP as an eligible VP environment for our study.

### Patient case

A single and anxious 59-year-old woman, unaware of her illness, was portrayed in a VP case story. She had lost her husband some years ago in a car accident and she was very worried about her sick and senile mother, as well as her adventurous and unpredictable son. She had been infected with tuberculosis (TB) 30 years before when she worked as a missionary in Africa. The TB infection remained latent for many years but became active recently in a time of emotional distress. She had general and local symptoms such as fatigue, weight-loss and cough.

### Participants

Thirty medical students from the sixth semester (clinical level) of the medical program at Karolinska Institutet were asked to voluntarily participate in this study, and all accepted. They were 13 men and 17 women; the median age was 24 years (Inter Quartile Range (IQR) 23–27)). Four of them were on assignment at the Danderyd University Hospital and the remainder at the Karolinska University Hospital. None of the participants had prior experience with the ISP system.

A consent form was signed by each of the participants before they started the VP session. The study was approved by the ethical committee at Karolinska Institutet.

### Method

Based on the aforementioned theoretical framework about emotional and experiential learning, we assumed that if the VP learning material would be perceived as emotionally and socially engaging, this would favor a lasting knowledge retention effect. For this reason, and in order to mainly focus on investigating the prerequisites for active and effective learning, we did not intend in this study to measure any cognitive learning outcome or any knowledge decay over time. Hence, an exploratory research approach with a user-centered design was considered for investigating the students’ interpersonal behaviors and socioemotional interaction exhibited during the Virtual Patient encounter. The investigation included what kind of emotions, feelings and moods could be evoked in a virtual patient interaction. Hence, we set up a study to explore affective learning elements (such as enjoyment, satisfaction, awareness, engagement, motivation, meaningfulness, etc.) during the VP sessions. The set-up focused also on the perception of patient presence and arousal induced by the interactive visual media. In an effort to establish these affective outcomes in relation to the actual cognitive performance, we employed a methodological triangulation (Larsen [36]; Sjöberg [37]).

#### Procedure

Pairs of students were assigned to solve a VP case on tuberculosis in the VP system. The VP scenario had been specifically designed with emotionally loaded video clips in order to investigate the framing salience of the computer-mediated learning experience among the participants.

There was no time limit for the students to complete the case. This allowed us to exclude time constraint as a potential confounder (i.e., stress factor) and to measure session length variance. A subset of the students was also videotaped during the whole VP session. One of the reasons to have the participants working collaboratively was to foster the thinking aloud process in order to ease the rating process of video observing verbal communication, perceptions and behaviors among the participants.

#### Data collection

The assessment of the collected data was articulated around mixed methods which consisted of a collection of student–VP interaction activity, video observational data, user experience questionnaire, and interview data to provide a holistic understanding of perceived feelings, expressed attitudes and observed behaviors during the learning experience. The multiple sources of data were based on the following collection methods (chronologically):

1. Interaction activity with the VP system was automatically registered in log files during the sessions for further quantitative analysis; it comprised a complete and detailed history of actions performed and decisions taken, including completion time, medical history taking, physical examinations performed and laboratory tests ordered, suggested preliminary diagnosis, confidence level (VAS scale) and diagnosis justification.

2. Video observational data was performed to assess the VP–student interaction and included both the affective and behavioral characteristics expressed by two students in a triad interaction (i.e., two students and one VP in our case). A coding scheme was constructed to assess the interactive modalities as well as the verbal and nonverbal interplay and 26 behavioral classes were defined (see Additional file 1 for detailed information). This scale was based on the most relevant constructs from the Roter’s Interaction Analysis System (RIAS) coding system (http://www.riasworks.com/index.html) when applied to a computer-mediated doctor–patient communication setting. A digital video camera captured facial expressions, body posture and gestures during the whole virtual patient encounter. Three independent raters screened the video recordings and then coded and annotated all student interactions with regard to verbal and nonverbal communication. We also assessed if the camera seemed to be obtrusive or not during the VP session. Observer agreement was then measured using Spearman’s *r* correlation coefficients. These scores were then corroborated with student perceptions, attitudes and performance scores, ascertained through the other collected data. Fourteen of the participants (i.e*.,* n = 7 sessions with 2 students per session; 9 females and 5 males) were videotaped. They had been initially offered to be videotaped and all of them accepted.

3. Questionnaire data: Exit questionnaires were administrated directly after the interviews. They contained questions regarding demographic data, self-reported IT proficiency, and an educational evaluation survey about the VP case. The rationale for administering the questionnaire after the interview, and not before, was to avoid receiving too spontaneous, and maybe not fully reflected and/or poorly articulated, answers from the respondents’ learning experience.

4. Semi-structured interviews with paired students (n = 15 interviews with pairs of students) were conducted in order to obtain data about students’ appraisal judgments with respect to their learning experience and attitudes to the VP encounter. A special emphasis was placed on students’ spontaneous reactions and feelings. The idea was also to gain an understanding of the affective dimensions of the social interaction among paired students, as well as between students and the VP.

## Results

### Log activity and gender-related outcomes

As shown in Table 1, the analysis of the log files revealed a discrepancy in completion time with regard to gender-related group distribution, favoring mixed groups in terms of time efficiency.

**Table 1:** Median values, inter quartile range (IQR) for session time and means of history questions based on gender-related group distribution

		**Session time (min)**		**History questions**				**Physical exam**	**Lab tests**
***Group***	***n***	**Median**	**IQR (p25, p75)**	**Mean no**	**% Biomedical**	**% Psychosocial**	**% Relevant**	**% Relevant**	**% Relevant**
Male–male	4	46	31-66	21	80	20	99	89.5	86.2
Male–female	5	36	29-52	18	83	17	100	90.6	95.8
Female-female	6	49	49-51	20	88	12	100	84	92.2
**Total**	**15**			**19.6**	**84**	**16**	**100**	**86**	**92**

In this study group the mean relevance of medical history questions for this patient case was 100%. The relevance for the physical examination and the selected laboratory tests was slightly lower, 86 and 92 respectively. These results reflect a realistic clinical encounter where a non-relevant medical history is less likely to occur than an exact relevance of for example laboratory tests.

Intriguingly, it was found that the percentage of psychosocial questions, regardless of the session length and group distribution, was higher for male students when compared with female students in this study setting (Table 2).

**Table 2:** Psychosocial questions’ ratios between gender-related groups

	**Ratio**	**SE**	**95% C.I.**		**P-value**
**Group comparison**			**Lower**	**Upper**	
Male-male/Male–female	1.61	0.228	1.03	2.52	0.037
Male–female/Female-female	1.71	0.254	1.04	2.82	0.034
Male-male/Female-female	2.76	0.233	1.75	4.35	<0.001

*Offset=Session Duration (min.).

Deviance/df=0.972.

In order to assess the ratio for the relative amount of psychosocial questions related to gender distribution, a Generalized Linear Model with a Poisson error distribution and a log link was applied. Log (Time) was used as an exposure variable (offset) in the analysis to adjust for the different amount of time the students used to interact with the VP. Thus, the outcome was the number of questions per minute. The model did not show any sign of overdispersion; deviance per degrees of freedom displayed a value of 1.2, which can be regarded as a good model fit.

### Video observation

Many observational variables were coded above the average, indicating positive affective learning outcomes in general as is shown in Table 3. Observed variables such as Overall Interaction Flow, Immersion Level, Consensus between Students, Interest/Attentiveness, and Responsiveness/Engagement showed rather high values, which indicates that the Virtual Patient encounter appears to have engaged the students affectively.

**Table 3:** Median values from observed affective and behavioral variables (*)

**Global observational variable**	Scale**	**Median all**	**Median normal screen**	**Median large screen**	**Median males**	**Median females**
Overall emotional atmosphere	*1-3*	**1.6**	1.0	1.0	1.0	1.0
Overall Interaction Flow	*1-3*	**2.2**	2.0	3.0^(+)^	3.0	2.0
Immersion Level	*1-4*	**3.0**	3.0	4.0^(+)^	3.0	3.0
Student mood state	*1-3*	**2.6**	3.0	3.0	3.0	3.0
Interpersonal Communication of Emotional States	*1-3*	**2.3**	2.0	3.0^(+)^	2.0	2.0
Consensus between students	*1-4*	**3.3**	3.0	3.0	3.0	3.0
Self-confidence level	*1-3*	**2.4**	2.0	3.0^(+)^	3.0	2.0
Communication Skills	*1-3*	**2.2**	2.0	3.0^(+)^	3.0	2.0
Attitude Towards Patient	*1-3*	**2.0**	2.0^(−)^	1.0	2.0	2.0
Patient's Presence	*1-3*	**1.6**	2.0	2.0	2.0	1.0
Students speak during patient answer	*1-4*	**1.5**	1.0	1.0	2.0	1.0
Interruption of patient answer	*1-4*	**1.2**	1.0	1.0	1.0	1.0
Anger/Irritation	*1-6*	**1.8**	2.0^(+)^	1.0	1.0	2.0
Anxiety/Nervousness	*1-6*	**1.6**	1.0	1.0	1.0	1.0
Dominance/Assertiveness	*1-6*	**4.0**	4.0	4.0	4.0	4.0
Interest/Attentiveness	*1-6*	**4.3**	4.0	5.0^(+)^	5.0	4.0
Friendliness/Warmth	*1-6*	**3.4**	3.0	4.0^(+)^	3.0	4.0
Responsiveness/Engagement	*1-6*	**4.1**	4.0	4.5^(+)^	4.0	4.0
Sympathetic/Empathetic	*1-6*	**3.0**	3.0	4.0^(+)^	3.0	3.0
Respectfulness	*1-6*	**3.5**	4.0	4.0	3.0	4.0
Hurried/Rushed	*1-6*	**1.6**	1.0	1.0	1.0	1.0
Body Lean	*1-4*	**2.5**	3.0^(−)^	2.0	2.0	3.0
Head Nod	*1-4*	**1.3**	1.0	1.5^(+)^	1.0	1.0
Hand gesture	*1-4*	**1.7**	2.0^(−)^	1.5	1.0	2.0
Eye contact/Eye gaze	*1-4*	**3.2**	3.0	3.0	3.0	3.0
Awareness/sensitivity to camera presence	*1-4*	**1.3**	1.0	1.0	1.0	1.0

*All median values are based on 14 students observed each by 3 independent raters.

**Scales are ranging from negative to positive.

^(+)^indicates a positive effect of larger screen.

^(−)^indicates a negative effect of larger screen.

Reliability of the coding system was assessed by computing an internal consistency test, which yielded a Cronbach’s alpha of 0.84.

The inter-rater reliability indicated low variation among raters which further indicates high reliability (intra-class correlation coefficient (ICC) was high: 0.84). There was no multiplicative interaction between the cases and the variables (Tukey’s test for non-additivity was found to be non-significant).

Among the global observation variables predictors were estimated for higher rated overall emotional atmosphere and immersion levels by computing the Spearman correlation coefficient (*r*). Overall emotional atmosphere was most strongly correlated with Interest/Attentiveness, followed by Responsiveness/Engagement and Eye contact/Eye gaze. Interpersonal Communication of Emotional States were the most strongly correlated with Immersion Level, followed by Head Nod), Communication Skills and Self-confidence.

The students were grouped in relation to the completion time in three categories; those with short completion time (31–36 minutes, 6 students), medium completion time (49–51 minutes, 6 students), and long completion time (87 minutes, 2 students). Concerning overall emotional atmosphere and interaction flow, consensus between students, communication skills and patient presence, the students with medium time had a significantly lower rating, particularly in patient presence, compared to those with short and long completion times. The students with a long completion time and two of the students with medium long completion time were sitting in a different location which may have had an impact on the results. More precisely, the completion time was found to be correlated negatively with increased Interest/Attentiveness (*r* = −0.79), followed by Overall Interaction Flow (*r* = −0.67), Level of Patient Presence (*r = −*0.68), and Communication Skills (*r* = −.58). In relation to those co-variations, the observed male participants showed a tendency to be more focused, with higher levels of Interest/Attentiveness (Mann Whitney U test: Mean Rank = 11.60 vs. 5.22, p = .004), Patient Presence (Mean Rank = 10.80 vs. 5.67, p = .029), and Overall Interaction Flow (Mean Rank = 10.70 vs. 5.72, p = .029) than female participants.

There were not many interruptions of the patient’s answers, possibly due to concentration effort (according to the raters’ observations and notes). Nonetheless the students’ apparent sympathetic/empathetic responses were averagely rated (median for Sympathetic/Empathetic = 3 on a 6-point Likert-type scale), indicating a somewhat less genuine behavior towards the virtual patient as opposed to what might be expected from a real patient encounter.

The co-variations of Attitude Towards Patient with other variables were also investigated by means of Spearman’s *r.* A strong association was found between Attitude Towards Patient and Screen Size (*r* = .84, *p* < .001), followed by Body Lean (*r* = .82, *p* < .001), Interpersonal Communication of Emotional States (*r* = .80, *p* = .001), Communication Skills (*r* = .68, *p* = .007), Immersion Level (*r* = .63, *p* = .016), and Interest/Attentiveness (*r* = .61, *p* = .020). These findings were also in line with the interview results.

Finally, the video camera did not seem to have been intrusive (median = 1.3). A rather unexpected reaction concerning the presence of the camera was that before commencing the VP session a few students asked if the VP was able to see them.

### Student evaluation

#### VP surveys

The students’ survey responses regarding perceptions, attitudes and learning preferences with the VP are presented in Table 4. Overall, we can observe that the majority of the participants were very positive concerning this kind of learning aid. The overall ratings compare favorably with those from a previous survey based on another ISP case (Courteille *et al.*[38]).

**Table 4:** Students’ spontaneous responses to the VP survey questions

Question (with free text response)*	**Thematic categories**	**Ranked frequency (%)**
1. What are your first reactions about ISP?	Enjoyable	75
	Educational	35
2. What is your opinion about training with this learning method?	Complement to other learning aids	30
3. How did you experience this way of solving a clinical problem?	Realistic	30
	Fun and enjoyable	25
	Instructive; rewarding	25
	Stimulating; challenging	15
4. What contributed to the realism of the VP case?	Patient speaking; images and video sequences of the VP	60
	Trustworthy case; real patient/person	45
	Physical Examination and laboratory tests	25
5. What is the value of motion picture with the voice of the patient?	High value	65
	Increased patients presence; life like; realistic	35
	Trustworthy; believable	15
	Engaging; stimulating; Inspiring; better focus	15
6. The direct feedback is important.		95
7. Do you have a previous experience with simulated or fictive communication?		25
8. How do you rate the ISP compared to paper-based cases?	More enjoyable/more fun	50
	At least as good as, or better	35
	Increased awareness/concentration	25
	More realistic	20
	More engaging/stimulating	20

*Representative statements/questions from a large survey.

We extracted thematic categories that arose from the analysis of free-text responses, and ranked them in the most frequently reported order. The ranking showed that the VP was experienced as realistic (30%), enjoyable (25%), and instructive (25%). The perceived realism was mainly attributed to the videofilmed patient (60%) as well as the trustworthiness of the case (45%). Students valued highly that the patient could “talk and move on the screen” (65%) and conveyed an increased sense of presence (35%). They overwhelmingly agreed on the importance of the feedback (95%). None of the aforementioned categories were reported in a negative manner. The VP system was mostly (50%) experienced as more fun/enjoyable than paper-based cases.

Table 5 presents the underlying “IT profile” for the participants, as well as their opinions regarding ISP as a possible virtual learning tool for applying medical knowledge (content validity). The latter was rated high (median = 4.5 on a 5 points Likert-type scale). Not surprisingly, participants valued computer simulations as important for promoting learning, and estimated their IT literacy as proficient. They were also well-inclined towards computer-supported collaborative work (somewhat more so for males). Male students reported slightly higher levels of interest for IT than females (Mann–Whitney U tests, mean rank = 19.15 vs. 12.71, p = .048).

**Table 5:** Student responses to the IT based survey

Question**	**Median**	**IQR**	**Males**	**Females**
1. Was the VP program designed for applying your knowledge?	4,5	4.0-5.0	5	4
2. Are computer simulations important for promoting learning?	4	3-4	3.5	4
3. Do you master IT-based learning tools?	4	3.75-4.25	4	4
4. When I use IT-support for learning I prefer to collaborate with others.	3	3-4	4	3
5. The use of IT is interesting	4	3-4	4	3
6. The use of IT is enjoyable	3	2-4	3	3
7. I feel fully concentrated when I use IT	3	2-4	3	3
8. Telepresence and Web***	1	1-2	2	1

**Five-point Likert-type scale (1=not at all, 5 =highly).

***Telepresence was based on the following 7 items (with a yielded Cronbach’s alpha coefficient of 0.83):

1. I forget about my immediate surroundings when I use the Web.

2. Using the Web often makes me forget where I am.

3. After using the Web, I feel like I come back to the "real world" after a journey.

4. Using the Web creates a new world for me, and this world suddenly disappears when I stop browsing.

5. When I use the Web, I feel I am in a world created by the Web sites I visit.

6. When I use the Web, my body is in the room, but my mind is inside the world created by the Websites I visit.

7. When I use the Web, the world generated by the sites I visit is more real for me than the "real world".

#### Interviews

Fifteen semi-structured interviews with all the study participants (n = 30) were undertaken. All students were interviewed in pairs (n = 15) with an average interview duration of 30 minutes. They reported their spontaneous feelings, opinions and attitudes directly after the VP session. A content analysis methodology was applied to the transcribed interviews. For triangulation purposes these outcomes were then related to the VP survey and to the actual performance of the trainee’s clinical interview.

Major themes that emerged from the analysis revealed that:

○ the trustworthiness and consistency of the VP case were highly regarded and facilitated maintaining focus on the simulated consultation,

○ the VP’s pedagogical design was beneficial for collaborative learning,

○ the conversational interface was experienced as a key factor for emotional engagement and knowledge retention,

○ the virtual patient was perceived as a real patient with real psychological concerns.

The interviewees described the patient case as trustworthy and they reported the story as being consistent and not fictitious. They highly valued the fact that the patient’s condition and situation unfolded in a manner similar to that in a real life consultation. Hence, most of them thought that the VP was a real patient and not an actor playing a role. However, some participants mentioned that they sometimes interviewed the VP in an inappropriate way because they were well aware of *“not conversing with a real person”*. Interestingly, all students anticipated that the case was a real patient despite the fact that she was portrayed by an “amateur”.

Many of the respondents emphasized the pedagogical importance of the interactive dialogue with the VP during medical history taking. They valued it as an effective way to activate and engage in the learning activity, as opposed to a passive interaction with a predefined “*drop-down menu listing and revealing all available questions”.* It was reported that they believed they would “recall better” when using a natural language based system such as ISP. It was advantageous that the interactive medical history of the VP case had been “fine-tuned” with caution during a prior pilot study, and very few negative comments due to dialogue failure were brought up. One student said *“the virtual patient just answered the questions that were asked and not more, as it has to be”.*

The vast majority of the students primarily perceived the VP as a meaningful and effective learning environment enabling them to *“solve clinical problems at one’s own pace”* and *“concentrate fully on the case”* without feeling the psychological pressure of *“managing every patient’s expectations”.* They also appraised it mainly as a tool for investigating a case and sharing understanding, rather than for skill or knowledge acquisition although a *“huge bunch of lab tests”* were available. Most of the students rated the VP system as very motivational and many also emphasized that the overall high interactivity, the free navigation in the system, as well as the extensive content library of medical data, simplified for them to apply their knowledge timely to solve the clinical case. They also felt that collaboration and discussions between peers benefited directly from the interactive features, and fostered shared understanding. Some groups used for instance the built-in pause function in order to temporarily *“put the VP in standby”* when current discussions became more intensive and/or required more thoughtful argumentation. Groups with mixed genders reported in the interviews that they perceived their collaborative interaction with higher degrees of flow and satisfaction compared to single-gendered groups. The data on mixed genders should however be analyzed with caution since the cohort is small and the results should be evaluated in a larger setting. Students described the minimalist instructional model of ISP as a way to more deeply stimulate and activate learning.

Overall, appraisals from the respondents conformed well to their written opinions (presented in the survey) and no student was found to present contradictory reports. All students but one mentioned that the video camera was not felt as intrusive and did not affect their concentration.

## Discussion

Research has shown that contextualized learning, feedback, and motivation are part of a process of achieving shared understanding (Hakkarainen *et al.*[39]). The combination of user acceptance, meaningfulness, deep engagement and emotional attachment has also positive and proven learning effects (Malone & Lepper [40]; Estrada *et al*. [41]). In this study, a vast majority of the participants expressed a positive consensus concerning the educational value of the VP learning environment, whose unfolding scenario was also perceived as taking place in an authentic and meaningful clinical context.

Our observations indicated that the VP environment did promote socioemotional interpersonal interaction with the virtual patient, and this in an appropriate and respectful manner. These behaviors are important to exhibit since there is reported a positive association (Weng *et al.*[42]) among doctor–patient relationship, patient trust and patient satisfaction. The study participants also demonstrated an adequate and relevant clinical case management as described earlier in Table 1.

Aligned with current research on computer-mediated communication skill training for physicians (see Roter *et al*. [43]), predictors of good emotional atmosphere in patient–doctor communication were not unexpectedly Interest/Attentiveness, Responsiveness/Engagement and Eye contact/Eye gaze. This knowledge and the mutual benefits, from a medical and human point of view respectively, should be highlighted and discussed with the medical students and preferentially with all who are involved in care of other human beings.

According to our interviews and video observations, the collaborative workspace of the VP system studied appeared to perform its social role by encouraging and creating favorable conditions for dynamic and engaged discussions fostering reflective learning and shared understanding. This confirms previous favorable observations on collaborative online learning with a VP (Bergin *et al*. [35]). The social interactions were observed under rather low levels of anxiety, which in its turn is a prerequisite for better learning conditions and learner performance.

In this study, we identified and measured the impact of a number of variables in the patient simulation that seem to be important to activate students’ learning as well as to motivate and engage them. We also investigated medical students’ affective reactions in the virtual encounter, as well as how they perceive the patient’s “mediated presence”. Such knowledge might be used for creating more effective and authentic experiences for emotional learning in medical education.

Subsequently, it was determined that the trustworthiness and the realism of this to some degree life-like situation, as well as the believability of the story and the performance of the actor (perceived by many participants as a real patient), played a key role in students’ appraisals. Research regarding synchronous interaction has suggested less overall interaction in text-based than in face-to-face communication (Lebie *et al.*[44]). In our case, the comprehensive and video-mediated medical history framework enabled an affective dimension in the student-VP interplay. Even though the video-observed sample was rather small, the social activity with the lifelike VP appeared to be both involving and immersive with satisfactory overall flow levels.

The interviews revealed that the advanced interaction modalities in the virtual clinical encounter eased learners in applying their knowledge to solve the clinical case. Nonetheless substantial differences were found in the students’ rapport with the VP and the flow of conversation. The low level of interruption of the virtual patient accords well with real patient–doctor communication. It is also interesting to report that mixed-gender groups (male–female) performed faster than male-only groups. In fact, the observation of the social interaction was reported with higher flows and satisfaction for the mixed-gender groups as opposed to the others. Mixed-gender groups might thus lead to a better synergy during a VP learning experience.

Liaw [45] claims that users having more extensive computer-related experience tend to perceive computer use and IT more positively. It is apparent from Table 4 that the participants’ rich IT experience might be a basis for their highly positive attitudes toward the value of simulation-based learning environments for solving clinical problems.

The slightly sharpened focus (i.e., heightened interest, attention and engagement) for men as opposed to women (Table 4) might partially be related to the self-reported higher levels of interest for IT among men.

Video-observation screening indicated that males seemed to be more attentive and more responsive listeners than females. This can be corroborated with the higher number of psychosocial questions they asked. This interesting result may partly be explained by the co-variation of IT-competence and positive engagement modes we measured, and placed in relation to higher flow states and patient’s presence levels. The finding is however derived from a small sample size where other factors may have had an impact as well, considering that females usually perform better than men in communication skills and with better empathic abilities (Van den Brink-Muinen *et al.*[46]).

In other words, this kind of computer-mediated and collaborative communication fosters measurable and valuable effects on interpersonal communication skills training, favoring mixed-gender groups in particular.

Not surprisingly, and conforming to communication research (Reeves *et al*. [47]), a larger screen (i.e. a larger patient face) appeared to have positive effects affecting in particular the immersion level, the emotional engagement, the completion time, and leading to reduced anxiety or nervousness. These findings are consistent with the media studies performed by Reeves and Nass [48], where they demonstrated that large faces on a screen could invade a person’s body space and induce more emotional arousal. Likewise, other media studies have also reported that presence factors contribute to heightened involvement, increased concentration, reduced cognitive load and, as a result, higher knowledge retention (Van Vliet & Specht [49]; Enlund [50]).

The quantitative results were also ascertained by the post-interview survey questionnaire emphasizing the positive effect of this experiential learning modality on student concentration, attention, motivation, and socioemotional interaction.

### Limitations

However, the small sample size (in particular for the video observation) might limit the interpretations of our findings, and we need to consider a larger study with even more patient cases. Rating of behaviors and nonverbal communication is highly subjective, and was not found to be an easy task. Although the internal consistency was considered as high, we want to stress the crucial need to recruit well-trained raters so as to guarantee the reliability of the video-coding process. Furthermore, it can be noted that 4 of the 14 videotaped participants were sitting in a different location (Danderyd hospital) and were overall rated lower by the observers, and needed a longer completion time. One possible explanation for this phenomenon might be that the students at this location were sitting in a somewhat noisy room in a hospital facility, whereas the other groups attended our department and were allocated a much quieter and better-equipped room.

### Future work

Although a more in depth process-oriented analysis of the studied interpersonal behavior and socioemotional interaction would lead to a more profound understanding of the participants’ socially shared motivation and emotions as well as the regulation strategies involved in a collaborative learning situation, we believe that the results and observations from the present study are very much relevant to serve as a basis for further design of virtual clinical encounters.

Due to the sample size and methods involved, our study aims and research questions have been partly but not fully addressed and we therefore intend to further develop our research methodology in order to achieve a more complete and conclusive holistic investigation. Further, since the present knowledge in medical education highlights the fictive encounter as a critical component in engaging VP-based learning environments (Roberts *et al*. [10]; Hubal *et al*. [51]; Cook & Triola [9]; Baños *et al*. [33]), it is consequently important to use, develop and evaluate technology in a meaningful way in order to to facilitate exploration and collaboration.

Important findings based on meta-analysis of modality effects have shown that different media can produce significant learning benefits [18,52,53]. We therefore also suggest further research to measure the emotional impact and level (sense) of presence with different mediated communication modalities on VPs. Experimental studies with VPs could, for instance, focus on avatars (virtual agents behaving in a human-like manner) vs. videofilmed actors, and/or standard shots vs. close-ups for assessing their respective contribution to learning, in relation to memory retention in particular.

## Conclusion

The results suggest that the VCE that was studied was perceived as a meaningful, intrinsically motivational learning environment, and has the potential to support the development of relevant and congruent interpersonal communication skills in trainees.

Satisfactory levels of social and emotional engagement were observed in learners. Design characteristics of the VCE environment such as the human actor mediated conversational interface and the presence of affectively evocative components played key roles in the learning experience.

It must be emphasized that by taking advantage of the socioemotional interaction between learners and the VP, these virtual learning environments offer to not only foster critical reflection skills or strategy-selection skills, but also to drive listening skills and nonverbal skills, to induce self-awareness and to challenge coping behaviours. These attributes in turn contribute to more salient beliefs and attitudes. The adoption of this more encompassing and engaging approach to clinical cases would also be particularly beneficial to increasing self-confidence in solving critical and emotionally distressing cases.

Our proposed innovative educational approach suggests a set of insights on how to design virtual clinical encounters with a focus on interpersonal communication skills. More generally, it will contribute to generate more insight in human affect in virtual psychosocial interviews.

Based on the findings in this study, we believe that innovative educational programs integrating professionalism, combined with developing caring and concerns for patients, in early medical education may lead to responsible clinical decision making, and facilitate the transfer to clinical practice.

## Competing interests

The authors declare that they have no competing interests.

## Authors' contributions

OC conceived of the study and designed it, performed the collection, analysis and interpretation of data, and drafted and revised the manuscript. AJ actively participated in acquisition of observational data and helped to revise the manuscript. LL participated in the design and coordination of the study, was involved in analysis and interpretation of data, and helped to draft and revise the manuscript. All authors read and approved the final manuscript.

## Pre-publication history

The pre-publication history for this paper can be accessed here:

http://www.biomedcentral.com/1472-6920/14/64/prepub

## Supplementary Material

Additional file 1 Virtual Clinical Encounter (Appendix). Click here for file
