# Supplementary material for: Interpersonal behaviors and socioemotional interaction of medical students in a virtual clinical encounter
Source: BMC Med Educ. 2014 Apr 1;14:64. doi: 10.1186/1472-6920-14-64 (PMC4229991; doi:10.1186/1472-6920-14-64)
Supplement: Additional file 1 — Virtual Clinical Encounter (Appendix). [file 1472-6920-14-64-S1.doc]

**Additional file 1**

| Global Observational Variable | Description/value | Student 1 | Comments | Student 2 | Comments |
| --- | --- | --- | --- | --- | --- |
| ***Student ID*** |  |  | |  | |
|  | | | | | |
| ***Overall emotional atmosphere***  Important: Common value here for both students! | Positive (engaged student, flow, fun, active discussion) |  |  | | |
|  | Neutral (unable to determine, holding back emotions (i.e. fail to be expressed)) |  |  | | |
|  | Negative (frustration, irritation, dominance of neg. comments, distance/barrier) |  |  | | |
| ***Overall interaction flow*** | Good |  |  |  |  |
|  | Satisfactory |  |  |  |  |
|  | Superior |  |  |  |  |
| ***Immersion level*** | Did not appear to be immersed at any time |  |  |  |  |
|  | Appeared to be immersed some of the time |  |  |  |  |
|  | Appeared to be immersed most of the time |  |  |  |  |
|  | Appeared to be immersed at all times |  |  |  |  |
| *Student mood state* | Bad |  |  |  |  |
|  | Neutral |  |  |  |  |
|  | Good |  |  |  |  |
| ***Interpersonal communication of emotional states*** | Limited |  |  |  |  |
|  | Normal |  |  |  |  |
|  | Intensive |  |  |  |  |
|  | | | | | |
| ***Consensus between students*** Overall agreement/consensus on tasks to perform for solving the case including formulation of history questions. | Little or no agreement |  |  |  |  |
|  | Agree some of the time |  |  |  |  |
|  | Agree most of the time |  |  |  |  |
|  | Agree all the time |  |  |  |  |
| ***Self-Confidence Level*** Evaluate the student's expressed level of self-confidence during the whole session. | Low |  |  |  |  |
|  | Medium |  |  |  |  |
|  | High |  |  |  |  |
|  | | | | | |
| ***Communication skills with patient during interview taking*** | Limited |  |  |  |  |
|  | Normal |  |  |  |  |
|  | Good |  |  |  |  |
| ***Attitude towards patient*** | Made judgmental comments, criticized patient; OR talked down to the patient |  |  |  |  |
|  | Made 1-2 comments with inappropriate affect |  |  |  |  |
|  | No judgmental comments; talked to patient as equal and offered praise/encouragement when opportunity arose |  |  |  |  |
| ***Patient's presence*** Expressed feeling of the patient being there, the virtual patient feels present. | Weak |  |  |  |  |
|  | Moderate |  |  |  |  |
|  | Strong |  |  |  |  |

| Global Observational Variable | | Description/value | | Student 1 | | Comments | | Student 2 | | Comments |
| --- | --- | --- | --- | --- | --- | --- | --- | --- | --- | --- |
| ***Student speaking during patient answer*** | | Little or not speaking | |  | |  | |  | |  |
|  |  | Some of the time | |  | |  | |  | |  |
|  |  | Most of the time | |  | |  | |  | |  |
|  |  | All the time | |  | |  | |  | |  |
| ***Interruption of patient answer*** | | Little or no interruption | |  | |  | |  | |  |
|  |  | Some of the time | |  | |  | |  | |  |
|  |  | Most of the time | |  | |  | |  | |  |
|  |  | All the time | |  | |  | |  | |  |
|  | | | | | | | | | | |
| ***Global Affect Ratings*** *Ratings are assigned on Likert scales from low to high (scale of 1-6)* | | | | | | | | | | |
| Anger/Irritation | | a "1" is assigned when there are no signs of the affect. | |  | |  | |  | |  |
| Anxiety/Nervousness | | a "1" is assigned when there are no signs of the affect. | |  | |  | |  | |  |
| Dominance/Assertiveness (confidently aggressive or self-assured) | | For these seven dimensions, a rating of "3" or "4" is considered "average" affect. This allows the coder to assign lower or higher ratings to reflect levels of low or high affect. For example, a "1" might be assigned when a student is markedly non-assertive; a "6" when the student seems especially dominant. | |  | |  | |  | |  |
| Interest/Attentiveness | |  |  |  | |  | |  | |  |
| Friendliness/Warmth | |  |  |  | |  | |  | |  |
| Responsiveness/Engagement | |  |  |  | |  | |  | |  |
| Sympathetic/Empathetic | |  |  |  | |  | |  | |  |
| Respectfulness | |  |  |  | |  | |  | |  |
| Hurried/Rushed | |  |  |  | |  | |  | |  |
|  | | | | | | | | | | |
| ***Body lean*** | Little or no forward body lean | |  | |  | |  | |  | |
|  | Some forward body lean | |  | |  | |  | |  | |
|  | Appropriate forward lean at most times | |  | |  | |  | |  | |
|  | Appropriate forward lean at all times | |  | |  | |  | |  | |
| ***Head nod*** | Little or no head nodding | |  | |  | |  | |  | |
|  | Some head nodding | |  | |  | |  | |  | |
|  | Appropriate head nodding at most times | |  | |  | |  | |  | |
|  | Appropriate head nodding at all times | |  | |  | |  | |  | |
| ***Hand gesture*** | Little on no hand gesture | |  | |  | |  | |  | |
|  | Some hand gesture | |  | |  | |  | |  | |
|  | Appropriate hand gesture at most times | |  | |  | |  | |  | |
|  | Appropriate hand gesture at all times | |  | |  | |  | |  | |
| ***Eye contact/Eye gaze*** | Little on no eye contact | |  | |  | |  | |  | |
|  | Some eye contact | |  | |  | |  | |  | |
|  | Appropriate eye contact at most times | |  | |  | |  | |  | |
|  | Appropriate eye contact at all times | |  | |  | |  | |  | |
| ***Awareness and/or sensitivity to camera presence*** | Little or no attention to camera | |  | |  | |  | |  | |
|  | Some of the time | |  | |  | |  | |  | |
|  | Most of the time | |  | |  | |  | |  | |
|  | All of the time | |  | |  | |  | |  | |
